# Research Advances in Molecular Mechanisms of Xylem Development in Horticultural Plants

**DOI:** 10.3390/plants15152401

**Published:** 2026-08-05

**Authors:** Lili Zhou, Menghao Wang, Shengjun Feng

**Affiliations:** 1 Horticulture Institute, Huzhou Academy of Agricultural Sciences, No. 768 Luwang Road, Huzhou 313000, China; zhoulili1437@126.com; 2 Ningbo Summit Advancement Discipline of Biological Engineering, College of Advanced Agricultural Sciences, Zhejiang Wanli University, Ningbo 315100, China; 17357783865@163.com

**Keywords:** horticultural plants, xylem, development

## Abstract

Xylem, a critical vascular tissue extensively distributed in stems and roots, plays indispensable roles in horticultural plant development. It facilitates water and mineral transport, provides mechanical support through secondary cell wall lignification, and precisely modulates ion homeostasis (e.g., Na^+^/K^+^ balance), thereby enhancing plant resilience to abiotic stresses. Consequently, xylem function directly impacts crop yield and quality. Recent breakthroughs in molecular biology have significantly advanced our understanding of the regulatory networks governing xylem development, including key transcription factors, hormonal signaling pathways (particularly auxin, cytokinin, and brassinosteroids), and their interactions with environmental cues. This review systematically summarizes current progress on the molecular mechanisms underlying xylem differentiation, secondary wall biosynthesis, and stress-responsive vascular adaptation in horticultural species. We further discuss emerging research frontiers, existing technical challenges, and prospective directions, aiming to provide a theoretical framework for genetic improvement and precision cultivation of horticultural crops.

## 1. Introduction

Horticultural plants, encompassing fruit trees, vegetables, and ornamental species, represent a cornerstone of global agriculture. According to the FAO Statistical Yearbook, the value of global primary crop production reached USD 2.9 trillion in 2022, with vegetables and fruit accounting for 19% and 17% of the total value, respectively. In China, horticultural crops rank second in cultivation area but first in total output value among all agricultural sectors, contributing to over half of the total output value of the planting industry [1]. According to the National Bureau of Statistics, China’s vegetable cultivation area reached 3.43 × 10^8^ mu by 2023, with total production reaching 8.29 × 10^8^ tons and average yield attaining 2415.26 kg/mu. These statistics underscore the rapid expansion and intensification of the horticultural industry [2].

Water, as the predominant component of plant biomass and a universal biological solvent, is fundamental to plant transpiration, photosynthesis, and mineral nutrient acquisition. Soil-derived essential elements are primarily absorbed by roots in ionic form dissolved in water, directly determining the productivity and quality of horticultural crops [3]. The root system serves as the primary interface for water and nutrient uptake, critically influencing water-use efficiency and biomass accumulation. Roots actively modulate hydraulic responses to environmental fluctuations [4] and coordinate shoot transpiration through systemic signaling. The stem functions as the central conduit for long-distance water transport, together with roots forming an integrated hydraulic continuum. A defining structural feature of both organs is the presence of specialized xylem tissues [5].

**Figure 1:**
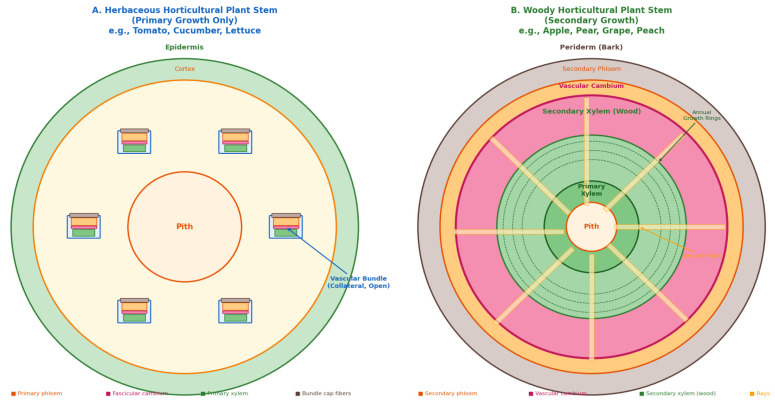
**Comparative Anatomy of Primary and Secondary Vascular Organization in Horticultural Plant Stems.** (**A**) Herbaceous horticultural plant stem (primary growth only). Typical of vegetable crops such as tomato (*Solanum lycopersicum*), cucumber (*Cucumis sativus*), and lettuce (*Lactuca sativa*). The stem contains discrete collateral, open vascular bundles arranged in a ring within the ground tissue (cortex). Each vascular bundle comprises, from the outside inward: bundle cap fibers (sclerenchyma providing mechanical support), primary phloem (conductive tissue for photoassimilate transport), a strip of fascicular cambium (a residual meristem with limited or no secondary activity in most herbaceous species), and primary xylem (water-conducting tissue). The primary xylem consists of protoxylem (first-formed, characterized by annular or spiral secondary wall thickenings) and metaxylem (later-formed, with reticulate or pitted walls). No continuous vascular cambium cylinder is present, and secondary xylem (wood) is absent. Water transport and mechanical support depend entirely on the primary vascular system. The center of the stem is occupied by pith (parenchymatous ground tissue). (**B**) Woody horticultural plant stem (secondary growth). Typical of fruit trees such as apple (*Malus domestica*), pear (*Pyrus* spp.), grape (*Vitis vinifera*), and peach (*Prunus persica*). Following the cessation of primary growth, fascicular cambium within vascular bundles and interfascicular cambium between bundles proliferate and connect to form a continuous vascular cambium cylinder. This lateral meristem produces secondary phloem outwardly and secondary xylem (wood) inwardly through periclinal divisions, driving radial thickening of the stem. The secondary xylem constitutes the bulk of the stem and is organized into annual growth rings (reflecting seasonal cambial activity), containing vessels (water conduction), fibers (mechanical support), and axial and ray parenchyma (storage and radial transport). Vascular rays (radial files of parenchyma cells) extend from the pith through the secondary xylem into the secondary phloem, facilitating lateral transport. The primary xylem is displaced toward the center by the accumulating secondary xylem and may be retained adjacent to the pith. The periderm (comprising cork cambium, cork cells, and phelloderm) replaces the epidermis as the outer protective tissue, together with the secondary phloem constituting the bark. Key distinction: In herbaceous stems, water transport depends on primary xylem within discrete vascular bundles; in woody stems, water transport depends on secondary xylem (wood), a continuous tissue produced by the vascular cambium.

The xylem constitutes the principal vascular tissue responsible for acropetal water transport and mechanical support in plants. It comprises multiple specialized cell types, including vessel elements, tracheids, fibers, axial parenchyma, and ray parenchyma, which together with phloem tissues form vascular bundles that establish an interconnected transport network (Figure 1) [6]. In angiosperm horticultural species, vessel elements represent the primary functional units for bulk water flow; their hydraulic efficiency is governed by anatomical characteristics including lumen diameter, pit membrane ultrastructure, and perforation plate architecture. Concurrently, xylem cells undergo extensive secondary cell wall deposition and lignification, which confer mechanical rigidity, enable vertical growth, and ensure architectural stability under mechanical loads and environmental stresses.

This review systematically synthesizes recent advances in xylem biology specific to horticultural plants, with particular emphasis on: (i) the molecular regulatory networks governing xylem cell fate determination, differentiation, and secondary wall biosynthesis; (ii) hormonal and transcriptional control of vascular development; and (iii) adaptive xylem plasticity in response to abiotic stresses. By integrating structural, physiological, and molecular perspectives, we aim to establish a comprehensive framework that bridges fundamental xylem biology with practical applications in high-quality cultivation and molecular breeding of stress-resilient horticultural varieties.

It is important to emphasize that the core molecular mechanisms governing xylem development—including hormone signaling, miRNA-mediated regulation, and transcription factor networks—have been predominantly elucidated in model and woody model species such as Arabidopsis, rice, and poplar. Considering the evolutionary conservation of these regulatory modules across vascular plants, the present review appropriately references findings from model organisms as a theoretical framework when direct experimental evidence from horticultural crops is not yet available. Importantly, the manuscript explicitly differentiates between mechanisms that have been experimentally validated in horticultural species and those inferred from model systems. A comprehensive summary of the evidence sources is provided in Table 1.

To ensure the reproducibility and balance of the references cited in this review, we performed a systematic literature search in databases including PubMed, GeenMedical, and Web of Science, and focused on English articles published within the past 15 years. Based on the summarized findings, we used “Horticultural plants; Xylem; Development” as the keywords for this review.

It is important to clarify the anatomical and developmental distinctions between primary and secondary xylem. Primary xylem differentiates from procambium during primary growth and is typically found in vascular bundles of herbaceous plants and young organs. Secondary xylem is derived from the vascular cambium during secondary growth, contributes to wood formation in woody perennials, and is the primary determinant of hydraulic conductivity and mechanical support in fruit trees and other woody horticultural crops. Throughout this review, we explicitly distinguish between these two xylem types. When evidence pertains specifically to primary xylem (e.g., vessel differentiation in tomato stems) or secondary xylem (e.g., cambium-derived xylem in apple or poplar), this distinction is clearly indicated. This review systematically synthesizes recent advances in xylem biology specific to horticultural plants, with particular emphasis on: (i) the molecular regulatory networks governing xylem cell fate determination, differentiation, and secondary wall biosynthesis; (ii) hormonal and transcriptional control of vascular development; and (iii) adaptive xylem plasticity in response to abiotic stresses.

Xylem formation encompasses multiple distinct developmental processes that are often conflated in the literature but must be distinguished for precise interpretation. These include: (i) primary xylem differentiation from procambium within vascular bundles, which occurs during primary growth and is typical of herbaceous organs; (ii) secondary xylem (wood) formation derived from vascular cambium during secondary growth, which drives radial thickening in woody perennials; (iii) vessel differentiation, including programmed cell death and cell wall remodeling; (iv) secondary cell wall biosynthesis, including cellulose, xylan, and lignin deposition; (v) lignification as a specific biochemical process within secondary wall formation; and (vi) stress-induced vascular remodeling, which involves adaptive changes in xylem anatomy under environmental perturbations. Throughout this review, we explicitly indicate which process each example refers to and avoid treating these processes as interchangeable. Diversity of vascular organization among horticultural plants: Horticultural plants encompass two major categories with fundamentally different vascular architectures. Herbaceous horticultural crops (e.g., tomato, cucumber, most vegetables) rely on primary vascular bundles for water transport and mechanical support, with limited or no secondary growth. Woody horticultural crops (e.g., apple, pear, grape, and ornamental trees) undergo extensive secondary growth, producing secondary xylem (wood) from the vascular cambium, which enables perennial growth and long-distance water transport. This review addresses xylem development in both categories, explicitly distinguishing between primary and secondary xylem throughout the text.

## 2. Research Progress on the Molecular Mechanism of Xylem Development

### 2.1. Integration of Phytohormone Signaling Networks in the Molecular Regulation of Xylem Development in Horticultural Plants

#### 2.1.1. Cambium-Derived Secondary Xylem Formation

As described in the Introduction, horticultural species vary in primary vs. secondary growth patterns. The vascular cambium is a critical meristematic tissue responsible for secondary growth in plants. Through periclinal divisions, it produces secondary xylem inwardly and secondary phloem outwardly, thereby driving the radial thickening of stems and roots. As a bipotent stem cell tissue, the differentiation fate of cambial cells directly dictates the developmental pattern of vascular tissues and the mechanical support capacity of plant organs [7]. Auxin is a key phytohormone regulating cambium development. At the signal transduction level, Auxin Response Factor 6 (ARF6) and ARF8 mediate auxin-dependent xylem formation. These transcription factors recognize and bind to auxin response elements (AuxREs), activating downstream target genes to initiate xylem differentiation programs [8]. Gibberellin (GA) indirectly modulates cambium activity by regulating polar auxin transport (PAT). During root development, GA upregulates the expression of PIN-family auxin efflux carriers, reshaping the auxin distribution gradient within vascular tissues [9]. In poplar and Arabidopsis, Exogenous GA treatment significantly promotes secondary xylem production, manifested as increased vessel element numbers, elevated proportions of xylem parenchyma, and expanded xylem cross-sectional area [10]. In the GA-biosynthesis-deficient mutant *ga1*, vessel and parenchyma cell numbers are markedly reduced—a phenotype that can be rescued by exogenous GA application [10].

#### 2.1.2. Primary Vascular Bundle Differentiation

Cambium stem cell activity is precisely regulated by a combination of phytohormones, peptide signals, and mechanical cues [10,11]. Beyond auxin, ethylene also plays a significant role in modulating wood formation. High-dose exogenous ethylene can significantly alter cambium growth, xylem morphogenesis, and cell differentiation patterns [12]. Yu et al. conducted a systematic investigation of ethylene’s role in vascular development using RhPMP1 (PETAL MOVEMENT-RELATED PROTEIN 1) RNAi and overexpression lines in Rosa hybrida. Phenotypic analyses revealed that RhPMP1-RNAi plants exhibited suppressed vascular bundle development, with reduced numbers of xylem, cambium, and phloem cells. In contrast, overexpression lines displayed opposing phenotypes, with enhanced vascular development—consistent across petal vascular bundles. Mechanistically, RhPMP1 overexpression upregulated auxin signaling proteins (e.g., RhAUX2), while RNAi lines downregulated these proteins. Chromatin immunoprecipitation confirmed that RhPMP1 directly binds to the RhAUX2 (auxin/indole-3-acetic acid 2) promoter. Immunolocalization studies demonstrated enhanced auxin signaling gradients from xylem to phloem in overexpression lines, contrasting with the attenuated signals in RNAi plants. Crucially, the ethylene precursor *ACC* (1-aminocyclopropane-1-carboxylic acid) strongly induced RhAUX2 expression in wild-type xylem cambium cells but failed to do so in RNAi lines. These results establish that ethylene promotes cambium activity through RhPMP1-mediated auxin biosynthesis and transport (a process central to secondary xylem formation) [12].

#### 2.1.3. Xylem-Mediated Transport of Nutrients and Metabolites

Shan et al. employed radioisotope labeling techniques to systematically investigate bicarbonate transport pathways in cucumber (Cucumis sativus) plants [13]. Experimental results demonstrated that bicarbonate absorbed by roots enters the xylem. Subsequently, amino acids, soluble sugars, and organic acids are transported via the xylem to the fruit exocarp and vascular tissues, directly influencing flavor quality. Further investigations revealed that cucumber NADP-dependent malic enzyme CsNADP-ME2 is predominantly localized in the fruit exocarp and vascular bundle system. Radioisotopic tracing and gas exchange analyses showed that CsNADP-ME2-overexpressing plants significantly enhanced the transport of soluble sugars and starch from roots to the fruit exocarp and vascular tissues through the xylem. In contrast, RNAi lines exhibited impaired transport, accompanied by reduced glycolysis-related gene expression and carbohydrate content [13].

#### 2.1.4. Stress-Induced Vascular Remodeling

Under drought conditions, tomato (*Solanum lycopersicum*) typically enhances its water transport capacity by increasing xylem vessel proliferation. However, excessive proliferation can lead to both exaggerated water loss and carbon resource depletion. Gibberellin (GA), a crucial phytohormone regulating vascular development, plays a significant role in this process, yet the precise mechanism by which its receptors (e.g., *GID1*) coordinate xylem development with water-use efficiency under water stress remained unclear prior to this study. Through CRISPR-Cas9-mediated genome editing, Illouz-Eliaz et al. successfully generated tomato gibberellin receptor mutants by knocking out *SlGID1a* and *SlGID1b* genes, creating the double mutant *gid1a/gid1b*. Their drought experiments yielded significant findings: while wild-type tomatoes exhibited substantial xylem vessel proliferation accompanied by noticeable leaf wilting under drought stress, the *gid1a/gid1b* double mutant displayed reduced xylem vessel density coupled with improved leaf water retention capacity and diminished biomass loss. Further physiological measurements revealed decreased stomatal conductance and reduced transpiration rates in the mutant plants. At the molecular level, the study demonstrated that the GA signaling repressor SlDELLA accumulated persistently in the mutants, directly suppressing the expression of key xylem development genes *SlVND6* and *SlVND7*, which serve as positive regulators of vessel differentiation. Chromatin immunoprecipitation (ChIP-qPCR) assays confirmed SlDELLA protein binding to the *SlVND7* promoter region. These findings collectively support three major conclusions: (1) under drought conditions, GA signaling through *SlGID1* receptors activates xylem proliferation, which while beneficial for short-term water transport, ultimately exacerbates water loss and carbon expenditure; (2) the mutants achieve “water-conserving drought resistance” by interrupting this signaling pathway; (3) SlDELLA functions as a molecular switch that integrates GA and ABA signaling to directly suppress *SlVND* expression, thereby maintaining equilibrium between vessel differentiation and stress response [14].

Cytokinins play a dual role in xylem development: they promote cambial cell proliferation on one hand, while antagonizing auxin to balance differentiation processes on the other. Their signaling is mediated through a three-tier phosphorelay system comprising histidine kinases (HKs), Arabidopsis histidine phosphotransfer proteins (AHPs), and response regulators (ARRs). Type-B ARRs function as transcription factors that directly activate genes associated with xylem development, whereas Type-A ARRs provide negative feedback to attenuate signaling intensity. Studies in tomato (*Solanum lycopersicum*) have shown that the concentration ratio of cytokinin to auxin determines the fate of procambial cells. A high CK/auxin ratio promotes phloem differentiation, while a low ratio induces xylem formation. Exogenous application of kinetin significantly increases xylem vessel density in tomato stems, although excessive application leads to disorganized vascular tissue patterning [14]. During graft union healing in cucumber (*Cucumis sativus*), cytokinins accumulate at the scion-rootstock junction, activating the expression of the *ARR12* homologous gene and facilitating vascular bundle reconnection. Silencing the *CsHK3* gene inhibits the regeneration of xylem cells in callus tissue [15]. In dwarfing apple rootstocks, attenuated cytokinin signaling is closely associated with restricted xylem development. Transcriptomic analyses reveal that the expression of *MdARR5* (a Type-A ARR) in the cambial zone of dwarfing rootstocks is significantly higher than in vigorous rootstocks, thereby suppressing the differentiation potential of the cambium toward xylem [15].

Brassinosteroids (BRs) primarily regulate xylem development through the BRI1 (receptor kinase)-BZR1/BES1 (core transcription factors) signaling module. BZR1 directly binds to the promoters of key transcription factors such as *VND7* and *MYB46*, activating programs for secondary cell wall synthesis and lignification. Moreover, BRs engage in extensive crosstalk with auxin and gibberellins to coordinately regulate cambial activity. In tomato, the BR-deficient mutant dwarf exhibits severe impairment in xylem development, characterized by reduced vessel cell numbers and incomplete cell wall thickening. Exogenous application of 24-epibrassinolide (24-eBL) rescues this phenotype and significantly upregulates the expression of *SlVND6*-like and *SlCESA7* (cellulose synthase) genes. BR signaling also modulates the composition of lignin monomers (S/G ratio) through the *SlBZR1-SlMYB308* regulatory module [16,17]. During the development of the fruit pedicel xylem in apple, BRs influence water transport capacity and fruit abscission. *MdBR* signaling modifies the plasticity of xylem cell walls by regulating the *MdXTH* (xyloglucan endotransglucosylase/hydrolase) gene family, thereby affecting vessel hydraulic efficiency [16,17,18].

Ethylene primarily functions in xylem development in response to mechanical stress and adversity in horticultural plants. In tomato, wounding or mechanical loading induces an ethylene burst, which subsequently upregulates the expression of *SlHB8* (an HD-ZIP III transcription factor) via *EIN3/EIL1* transcription factors, promoting the formation of reaction xylem to enhance structural support [19]. In apple, the peak of ethylene production during late fruit development coincides with the gradual occlusion of xylem vessels in the fruit stalk, which may be part of the hydraulic isolation mechanism associated with fruit maturation and abscission [20].

Jasmonic acid (JA) is more prominently involved in defense-related xylem responses induced by injury and pathogen infection. For example, following pathogen attack in tomato, JA signaling rapidly activates *SlMYC2*, thereby promoting the lignification of parenchyma cells surrounding vessels and the formation of tyloses to block pathogen spread [21].

Under drought stress, abscisic acid (ABA) induces narrower xylem vessel diameters and increased vessel frequency to reduce the risk of embolism. In apple rootstocks, ABA regulates stress-associated lignin deposition by activating the *MdMAPK6-MdMYB96* cascade [22].

### 2.2. MicroRNA-Mediated Molecular Networks Orchestrating Xylem Development in Horticultural Plants

Species considerations for miRNA studies in xylem development: Most miRNA-mediated regulatory mechanisms of xylem development have been discovered in model species (e.g., *Arabidopsis thaliana*, *Medicago truncatula*, poplar, rice, maize) due to their advanced genetic tools and well-annotated genomes. Where direct evidence from horticultural crops is lacking, we cite model species findings as a theoretical framework. This is justified because core miRNA-target modules—such as *miR165/166*-HD-ZIP III and *miR319*-TCP—are highly conserved across vascular plants, as demonstrated by phylogenetic and functional studies. However, direct functional validation in horticultural species remains a priority for future research. Throughout this section, we explicitly indicate whether each finding originates from model species or horticultural crops and discuss the predicted conservation where applicable.

MicroRNAs (miRNAs) mediate post-transcriptional gene silencing through sequence-specific recognition of complementary motifs within the 3′-untranslated regions (3′-UTRs) of target mRNAs, directing transcript cleavage or translational repression [23]. This regulatory modality enables precise spatiotemporal control of xylem developmental programs, spanning vascular cambium stem cell maintenance, xylem cell fate specification, secondary cell wall (SCW) biogenesis, and programmed cell death (PCD) [23]. The resulting miRNA-target regulatory networks form intricate, multi-layered architectures that integrate developmental cues with environmental signals.

Most plant miRNAs are encoded as single entities within miRNA precursors. These miRNAs post-transcriptionally regulate a novel family of transcription factors associated with nodule development, namely the class III homeodomain-leucine zipper (HD-ZIP III) genes. In *Medicago truncatula*, Overexpression of *MIR166* reduces the number of symbiotic nodules and lateral roots, and induces ectopic vascular bundle development in these transgenic roots [24]. *miR857* is involved in regulating lignin content, thereby influencing the morphogenesis of secondary xylem. Furthermore, under low-copper conditions, *miR857* is activated by SQUAMOSA PROMOTER BINDING PROTEIN-LIKE 7 (SPL7). Collectively, these findings reveal the regulatory role of *miR857* in secondary growth of vascular tissues in Arabidopsis and elucidate a unique control mechanism for secondary growth that operates in response to copper deficiency via *miR857* expression [25].

In poplar (*Populus alba × P. glandulosa*), Mutation of *PagMYB31* resulted in a reduced number of cambial cell layers, enlarged fusiform initials, ray initials, vessels, fibers, and ray cells, as well as enhanced secondary cell wall thickening in xylem cells, indicating that *PagMYB31* positively regulates cambial cell proliferation while negatively regulating xylem cell expansion and secondary cell wall biosynthesis. *PagMYB31* suppresses xylem cell expansion and secondary cell wall thickening by directly repressing genes encoding cell wall-modifying enzymes and transcription factor genes that activate the entire secondary cell wall biosynthetic program, respectively [26].

In *Populus trichocarpa*, Overexpression of PtrSCZ1 or its homolog *PtrSCZ3* (OE-*PtrSCZ1*, OE-*PtrSCZ3*) led to enhanced cambium activity, increased stem diameter, and a larger proportion of xylem. In contrast, CRISPR-based mutants of *PtrSCZ1* and *PtrSCZ3* exhibited opposite phenotypes compared with the overexpressing plants. These results indicate that *PtrSCZ1* and *PtrSCZ3* act redundantly to promote cambium activity and secondary growth, thereby increasing radial growth in *Populus trichocarpa*. Moreover, both overexpression and knockout of *PtrSCZ1* and *PtrSCZ3* significantly altered the expression levels of key cambium regulators (including *PtrWOX4a*, *PtrWOX4b*, *PtrWOX13a*, *PtrPXYa*, *PtrVCM1* and *PtrVCM2*) and disrupted the expression of cell wall-related genes. These findings suggest that *PtrSCZ1* and *PtrSCZ3* may regulate cambium division activity by modulating these key cambium-associated transcription factors involved in wood formation [27].

In Arabidopsis, the NAC family transcription factor *VND7* serves as a core regulatory protein controlling the differentiation process of xylem vessels. Experimental evidence indicates that introducing the *VND7* gene rescues the secondary wall formation defects in the inflorescence stem fiber cells of the nst1 nst3 double mutant, and also restores the normal expression level of the *NST3* gene [28]. The secondary wall-associated NACs from rice and maize are capable of complementing the secondary wall thickening defects observed in the Arabidopsis snd1 nst1 double mutant. When overexpressed in Arabidopsis, *OsSWNs* and *ZmSWNs* are sufficient to activate multiple secondary wall-associated transcription factors and secondary wall biosynthetic genes, leading to ectopic deposition of cellulose, xylan, and lignin. Furthermore, we found that the rice and maize MYB transcription factors *OsMYB46* and *ZmMYB46* serve as functional orthologs of Arabidopsis *MYB46/MYB83*. Overexpression of these genes in Arabidopsis is sufficient to activate the entire secondary wall biosynthetic program [29]. To investigate the function of POPCORONA during secondary growth, we used transgenic poplar (Populus) plants expressing either a miRNA-resistant version of POPCORONA or a synthetic miRNA designed to target POPCORONA. Transgenic lines were compared with wild-type control plants in terms of overall morphology, histology, and gene expression patterns. Knockdown of POPCORONA via the synthetic miRNA resulted in abnormal lignification in pith cells, whereas overexpression of the miRNA-resistant POPCORONA led to delayed lignification of xylem and phloem fibers during secondary growth [30]. Overexpression of *PbXND1* in pear leads to a dwarf phenotype with defective xylem development, while *PbTCP4* promotes xylem development in roots [31] (Table 2).

**Figure 2:**
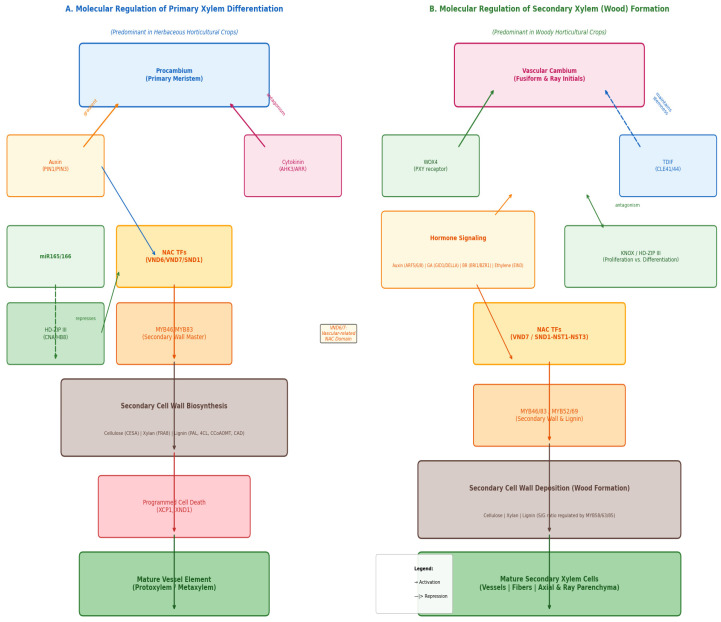
**Comparative Molecular Regulatory Mechanisms of Xylem Development.** This schematic illustrates the multi-layered molecular mechanisms governing xylem differentiation, integrating transcriptional control, hormone/peptide signaling, secondary cell wall biosynthesis, programmed cell death, and epigenetic regulation. Core Transcriptional Regulation. *NAC* master switches (*VND/SND*) activate *MYB46/83* to drive secondary cell wall (SCW) deposition and lignin biosynthesis, while XND1 negatively regulates this cascade. HD-ZIP III and KNOX families antagonistically balance cell differentiation versus proliferation. Hormone and Peptide Signaling. Auxin (via *ARF5-HB7/8*), cytokinin, and ethylene (via HD-ZIP I) coordinately regulate vascular patterning. CLE41/44-TDIF signaling through PXY receptor inhibits differentiation and promotes proliferation to maintain cambium stemness, counterbalanced by *EPFL4/6*-mediated cambium activation. Secondary Cell Wall Formation. Phenylalanine-derived lignin monomers (synthesized via PAL, 4CL, CCoAOMT, CAD) polymerize with cellulose microfibrils and hemicellulose to form rigid, hydrophobic SCW. Programmed Cell Death. Vacuolar hydrolytic enzyme release triggers cytoplasmic degradation, generating hollow, lignified functional vessels for water transport. Epigenetic and miRNA Fine-Tuning. *miR165/166* targets HD-ZIP III; *miR408/miR163* exhibit antagonistic regulation. DNA methylation at promoters transcriptionally silences key developmental genes. Legend: Red, NAC; green, HD-ZIP III; blue, KNOX; orange, enzymes/genes; brown, lignin; light blue, cellulose; green, hemicellulose. Solid arrows, activation; blunt lines, inhibition; bidirectional arrows, antagonism; zigzag lines, miRNAs.

The expression of multiple *GRF* genes is regulated by *microRNA396* (*miR396*), and the GRF-*miR396* regulatory module appears to be central to many of these processes [32,33] (Figure 2).

**Table 2:** Transcriptional factors related to xylem development and their upstream microRNA regulatory networks.

miRNA	Target Gene	Species	Organ/Tissue	Xylem Process	Evidence Type	Reference
miR165/166	HD-ZIP III (CNA1, CNA2, HB8)	*Medicago truncatula*	Root	Primary xylem differentiation	Overexpression in model species	[24]
miR165/166	POPCORONA (HD-ZIP III)	*Populus trichocarpa*	Stem (secondary growth)	Secondary xylem formation	Transgenic poplar	[30]
miR164/319a	VND7, NST1, NST2, SND1	*Arabidopsis thaliana*	Stem	Vessel differentiation/Secondary wall thickening	Mutant complementation	[28]
miR319	TCP	*Populus alba × glandulosa*	Stem	Secondary xylem development (salt stress)	Transgenic poplar	[34]
miR319	TCP	*Solanum lycopersicum*	Leaf, stem	Primary vascular development	Transgenic tomato	[35]
miR396	GRF	*Arabidopsis thaliana*	Multiple tissues	Secondary growth (dwarfing)	Overexpression	[32,33]
miR857	LAC (laccase)	*Arabidopsis thaliana*	Stem xylem	Secondary cell wall lignification	Overexpression	[25]

## 3. Research on the Mechanism of Xylem Response to Abiotic Stress

### 3.1. Responses of Xylem and Barrier Tissues to Drought Stress

Root barrier mechanisms and xylem water supply: In addition to direct regulation of xylem development, plants modulate water transport under drought through root-specific barriers such as suberin deposition. The following discussion focuses on how these root barrier mechanisms influence xylem water supply and whole-plant drought tolerance.

During evolution, plants have developed sophisticated mechanisms for cell type differentiation to integrate environmental signals with developmental processes. Specialized cell types enhance plant resilience to abiotic stresses by participating in the transport of minerals, nutrients, and water [3,36]. Two principal diffusion barriers exist in plants: suberization and lignification, with suberization showing stronger responsiveness to nutrient deficiency [36]. Suberin, a secondary metabolite composed of phenolic compounds, glycerol, and fatty alcohols, acts as a protective barrier in roots, regulating the flow of water, solutes, and gases [36]. Suberin deposition in the endodermis creates a hydrophobic barrier that controls water and ion entry into the xylem, thereby influencing xylem sap composition and hydraulic function. While suberin is primarily localized in the endodermis in most plants, it is found in the exodermis of specific species like tomato [37]. The *esb1* mutant in *Arabidopsis* exhibits enhanced drought tolerance due to increased suberin deposition without altered lignin content, and grafting with *esb1* rootstocks improves drought resistance in scions [38]. These findings highlight suberin as a root barrier mechanism that affects xylem water supply, rather than a direct component of xylem development. The hydraulic resistance imposed by suberized root layers directly influences xylem sap tension and overall plant water status, thereby linking root apoplastic barriers to the hydraulic performance of the xylem. In horticultural crops like tomato, gibberellin treatment substantially promotes suberin deposition in roots [37]. Molecular analyses indicate that multiple MYB transcription factors redundantly regulate the expression of suberin biosynthesis genes and their accumulation [39]. CRISPR-Cas9-generated tomato MYB mutants exhibit reduced suberin accumulation and diminished responsiveness to gibberellin treatment. These mutants display significantly lower drought tolerance metrics (e.g., stem water potential, relative water content, transpiration rate, stomatal conductance) and reduced suberin precursor levels compared to wild-type plants, confirming the critical role of suberin in drought resilience [39].

Plant laccases are key enzymes involved in the polymerization of lignin macromolecules. Previous studies have reported that the poplar *LAC17* gene is localized in the secondary cell walls of stem xylem and guard cells, where it functions as a critical regulator of lignin polymerization. In poplar lines overexpressing *PtrLAC17*, lignin content was significantly increased in the xylem cell walls, whereas opposite results were observed in gene knockout lines. Furthermore, knockout plants exhibited larger stomatal apertures and reduced drought tolerance, while overexpression lines showed smaller stomatal apertures and enhanced drought resistance. These findings suggest that increased lignin deposition in guard cell walls restricts stomatal opening, thereby improving plant drought tolerance [40].

Tyrosine decarboxylase (*TyDC*) catalyzes the conversion of tyrosine to tyramine and plays important roles in secondary metabolism, plant development, and responses to environmental stress. It has been reported that poplar *PaTyDC4* is involved in drought stress responses. Overexpression of this gene leads to increased ratios of xylem to phloem width, enlarged vessel cell areas, and enhanced lignin accumulation in stems. Furthermore, compared with wild-type plants, the overexpression lines exhibit significantly improved drought tolerance, indicating that *PaTyDC4* promotes xylem differentiation and lignin deposition during secondary growth, thereby contributing to enhanced plant resilience under drought conditions [41].

### 3.2. Physiological and Anatomical Adaptive Mechanisms of Plants to Salt Stress

Salt stress is a prevalent abiotic stress factor that severely restricts the growth, development, and expression of desirable traits in horticultural plants, significantly compromising their yield and quality [42,43,44,45]. The detrimental effects of salt stress on horticultural plants primarily manifest as osmotic stress, ion toxicity, and secondary stress [45,46]. In poplar, salt stress induces anatomical changes in xylem, including reduced vessel lumen diameter and altered fiber cell wall thickness, which are associated with the formation of a novel type of ‘pressure wood’ (Janz et al., 2012 [47]). In angiosperms, salt stress generally leads to narrower vessels and increased vessel frequency, which reduce cavitation risk but may compromise hydraulic conductivity [46]. MicroRNAs (miRNAs), a class of non-coding RNAs that regulate gene expression via complementary targeting [47,48], include the highly conserved *miR319* in horticultural plants [48,49]. Its overexpression induces marked changes in leaf morphology and curvature in tomato [50]. Notably, in tomato (*Solanum lycopersicum*), *miR319* also modulates vascular system development in horticultural plants by targeting TEOSINTE BRANCHED/CYCLOIDEA/PROLIFERATING CELL FACTORS (*TCP*) genes, which play critical roles in plant abiotic stress responses [51,52]. Overexpression of miR319 has been confirmed to reduce *TCP* protein abundance and secondary cell wall thickness in stems [51]. Given the intrinsic link between stem development and salt tolerance in horticultural plants, this mechanism indirectly affects their salt stress resilience (Stem development, particularly vascular differentiation and secondary cell wall thickening, contributes to salt tolerance by regulating ion transport and mechanical stability under stress). In poplar, salt stress significantly upregulates *miR319a* expression in stems. Functional validation using miR319a-overexpressing (OE) and *miR319a*-suppressed (MIMIC) transgenic lines under salt stress demonstrated superior growth in *miR319a*-OE plants compared to wild-type and MIMIC lines, indicating enhanced salt tolerance through *miR319a* overexpression. In horticultural plant stress responses, Na^+^/K^+^ homeostasis is pivotal [53,54,55]. Salt-stressed *miR319a*-OE plants exhibited significantly elevated Na^+^/K^+^ ratios, whereas MIMIC lines showed the opposite trend. High-throughput transcriptomic sequencing and Gene Ontology (GO) analysis of wild-type and transgenic poplars revealed that differentially expressed genes predominantly clustered into two categories: salt stress response pathways and xylem development processes. *PagSKOR1-b*, encoding a K^+^ long-distance transport protein, showed salt-induced upregulation with the highest expression in *miR319a*-OE and the lowest in MIMIC plants. Analysis of xylem cell wall development-related genes identified *PagMYB52*, a transcription factor regulating secondary cell wall biosynthesis, as upregulated in *miR319a*-OE and downregulated in MIMIC plants under salt stress. *PagMYB52* is essential for xylem vessel formation, as is *PagXCP1* (Xylem Cysteine Protease 1), another xylem development gene displaying elevated expression in *miR319a*-OE lines and reduced levels in MIMIC plants. Anatomical analysis of two-month-old stem cross-sections under salt stress revealed reduced cambial layers, widened secondary xylem, increased vessel and fiber cell numbers, and thinner secondary walls in *miR319a*-OE plants. These findings demonstrate *miR319a*’s critical role in regulating secondary xylem development under salt stress. Furthermore, miR319a enhances poplar salt tolerance by increasing vessel quantity and lumen area, mechanistically linked to upregulated expression of *PagHKT1;2* (High-affinity K^+^ Transporter 1;2) and *PagSKOR1-b* (Shaker-like K^+^ Outward Rectifier 1-b) [55]. These results conclusively validate the pivotal role of xylem development in horticultural plant salt tolerance [55].

Glutathione S-transferases (GSTs) are a class of multifunctional proteins in plants that play critical roles in regulating growth and development, synthesizing secondary metabolites, and responding to abiotic stresses. A research team led by Hu et al. revealed that Salt stress significantly induces the expression of the *SlGSTU43* (Glutathione S-Transferase U43) gene in the roots of wild-type tomato (*Solanum lycopersicum* cv. Ailsa Craig, AC) [34]. Functional validation using *SlGSTU43*-overexpressing lines and slgstu43 mutants demonstrated enhanced salt tolerance in overexpression plants and hypersensitivity in mutants. Transcriptomic (RNA-seq) analysis showed that *SlGSTU43* overexpression significantly alters the expression profiles of lignin biosynthesis-related genes. Further investigations identified a protein–protein interaction between *SlGSTU43* and *SlCOMT2* (Caffeic acid O-MethylTransferase 2), a key enzyme in lignin biosynthesis. This interaction enables *SlGSTU43* to regulate lignin content and promote plant growth under Salt stress. Mechanistic studies revealed that transcription factors *SlMYB71* and *SlWRKY8* form a transcriptional complex through protein interaction, specifically binding to the *SlGSTU43* promoter to activate its expression. This study elucidates the *SlMYB71-SlWRKY8-SlGSTU43-SlCOMT2* regulatory pathway: upstream transcription factors synergistically activate *SlGSTU43* expression, and its encoded protein interacts with *SlCOMT2* to enhance lignin biosynthesis, thereby improving tomato salt tolerance. These findings not only uncover novel roles of GST family proteins in plant stress adaptation but also provide critical insights into the molecular network linking lignin metabolism and salt stress responses [34].

Under salt stress, the two deciduous tree species/types with high wood density (HWD) and low wood density (LWD) exhibited distinct xylem anatomical phenotypes. The HWD group showed relatively small changes in vessel diameter under salt stress, whereas the LWD group exhibited a significant reduction in vessel diameter. Additionally, the LWD group had higher xylem water potential, while the HWD group demonstrated stronger salt stress tolerance [56].

Under salt stress, the stem height of Medicago sativa plants is reduced, and the number, length, and diameter of internodes are decreased. In addition, NaCl treatment increases the number of lignified phloem fibers and enhances their cell wall thickness. In the lower internodes of treated plants, the secondary xylem contains fewer vessels, while the number of lignified fibers increases. Comparative analysis reveals that the variations in secondary derivative distribution, the intensification of cell wall thickening, the lignification of phloem and xylem fibers, as well as the damage to the phloem complex, are more pronounced in the lower internodes than in the upper internodes. These findings illustrate the occurrence of altered secondary derivative distribution, aggravated cell wall thickening, lignification of phloem and xylem fibers, and injury to the phloem complex under salt stress [35].

## 4. Xylem-Mediated Improvement of Agronomic Traits

The agronomic traits discussed in this section—including lodging resistance, fruit flavor quality, disease resistance, and dwarfing—are not isolated phenomena but rather phenotypic manifestations of the stress-adaptive and developmental mechanisms detailed in Section 3. The xylem, as a central hub integrating hydraulic transport, mechanical support, and long-distance signaling, plays a pivotal role in translating these molecular and physiological processes into agronomically relevant outcomes. In the following subsections, we examine how xylem-mediated mechanisms contribute to each of these traits, explicitly linking them to the stress responses covered in Section 3.

### 4.1. The Xylem Provides Plants with the Ability to Resist Lodging

As illustrated in Table 3, xylem developmental processes—including cambium activity, vessel differentiation, secondary wall thickening, and stress-induced remodeling—directly influence lodging resistance, plant architecture, fruit quality, disease resistance, and abiotic stress tolerance. The following subsections elaborate on each of these connections. As a critical mechanical support tissue in plants, xylem development directly determines lodging resistance, a trait particularly vital in floricultural crops as it influences yield and cut-flower quality [51,57,58,59,60]. Studies have demonstrated the central regulatory role of SPL2 (squamosa promoter binding protein-like 2) transcription factors in stem vascular development [61]. Tang et al. systematically characterized the function of PlSPL2 in herbaceous peony (*Paeonia lactiflora*) [61]. Subcellular localization confirmed its nuclear localization, while tissue-specific expression profiling revealed predominant expression in stems. PlSPL2-RNAi lines exhibited enhanced stem strength, enlarged vessel lumen diameter, and increased xylem layers, confirming its role in suppressing xylem development. Heterologous overexpression in *Nicotiana benthamiana* further validated this function, as PlSPL2-OE plants displayed reduced mechanical strength, impaired xylem development, and narrowed vessel lumina. Collectively, these results establish PlSPL2 as a negative regulator of xylem development that ultimately compromises peony quality and yield. Xu et al. functionally dissected another SPL family member, PlSPL14 (squamosa promoter binding protein-like 14), in peony [61]. Phylogenetic and expression analyses revealed its high expression in stems, peaking during rapid elongation and secondary thickening phases. In situ hybridization localized *PlSPL14* specifically to stem internode cells. Functional studies showed that PlSPL14-OE plants exhibited thickened stems, elevated lignin content, and enhanced lodging resistance, whereas RNAi lines displayed opposing phenotypes. Cytological observations revealed disorganized vessel cell arrangement in RNAi lines, while OE lines showed intensified lignification and thickened cell walls in vascular bundles. Mechanistically, PlSPL14 directly binds and activates promoters of phenylpropanoid pathway genes (*CCR2*, *COMT1*). Hormonal assays demonstrated GA-mediated suppression and BR-induced promotion of *PlSPL14* expression, uncovering hormonal crosstalk in xylem development [62].

Liu et al. investigated the role of the tomato HD-Zip transcription factor *SlHB8* (*Homeobox 8*) in vascular regulation [63]. Expression profiling highlighted its enrichment in stem vascular tissues, particularly the cambium and xylem. Functional analyses revealed that *SlHB8*-overexpressing (OE) lines exhibited slender stems, reduced bending resistance, and decreased lignin content, whereas knockout lines displayed thickened stems, enhanced lignification, and improved lodging resistance. Anatomical studies showed impaired vascular development and thinner vessel walls in OE lines, contrasted by thickened secondary walls and orderly vessel alignment in knockout lines. Molecular dissection confirmed that *SlHB8* suppresses lignin biosynthesis by binding to L1-box cis-elements in the promoters of *CCR1* (Cinnamoyl-CoA Reductase 1) and *PAL2* (Phenylalanine Ammonia-Lyase 2). Transcriptomics revealed systemic downregulation of phenylpropanoid pathway genes in OE lines. Hormonal profiling linked reduced IAA and elevated GA3 levels in OE lines to auxin signaling antagonism, elucidating *SlHB8*′s role in modulating lignification and lodging resistance [63].

In fruit tree cultivation, spur-type apple varieties are highly valued for their compact architecture and space-efficient growth, traits that are governed by internode length—a parameter directly influenced by xylem and vessel development [64]. Gao et al. identified auxin-leucine resistant hydrolase *MdILL6* (IAA-Leucine Resistant-Like 6) in spur-type apples [65]. MdILL6 is highly expressed in shoot tips and stems. Overexpression of *MdILL6* (*MdILL6*-OE) caused shortened internodes, suppressed xylem development, reduced vessel number and lumen diameter, and downregulated genes related to cell elongation. Exogenous methyl jasmonate treatment further corroborated its inhibitory effect on xylem development. Integrated analyses demonstrated that *MdILL6* regulates xylem cell differentiation, modulates vessel development, and integrates phytohormone signaling to shape apple tree architecture. These findings advance the molecular understanding of plant form regulation in horticultural species [66].

### 4.2. Xylem Development Affects the Flavor of Horticultural Crops

Beyond its structural roles, xylem function also influences fruit and vegetable quality through long-distance transport of metabolites—a process intimately linked to the stress-responsive vascular physiology covered in Section 3.1 and Section 3.2.

Horticultural plants play a significant role in China’s agricultural production, with vegetable crops experiencing particularly high market demand. This makes the cultivation of high-quality horticultural varieties a critical objective.

Ahmed and Khadr et al. comprehensively explored the effects of indole-3-butyric acid (IBA) on carrot (Daucus carota) taproot development [67]. IBA treatment significantly promoted radial expansion while suppressing longitudinal growth in carrot seedlings. Cytological analyses revealed enhanced lignin deposition, thickened cell walls, and increased numbers of lignified cells in IBA-treated roots. Biochemical assays confirmed elevated lignin monomer content and intensified cellulose-lignin crosslinking in IBA-treated groups. Transcriptomic profiling further demonstrated IBA-induced upregulation of phenylpropanoid pathway genes, lignin biosynthesis genes, and peroxidase genes. Notably, excessive IBA application caused hyper-lignification, which adversely affected carrot edibility. This study provides crucial insights into the molecular mechanisms by which phytohormones regulate lignification to shape horticultural crop quality [67].

### 4.3. Research on the Mechanism of Xylem Affecting Plant Resistance

Disease resistance mediated by xylem lignification represents a specialized case of the stress-induced vascular remodeling discussed in Section 2.1.4, where lignin deposition serves as both a physical and chemical barrier against vascular pathogens.

Tomato bacterial wilt, caused by *Ralstonia solanacearum*, is a devastating soil-borne vascular disease. The pathogen invades host plants through root wounds, colonizes the vascular system, and spreads upward, ultimately leading to vascular tissue necrosis and plant wilting. Wang et al. investigated the specific resistance mechanisms of tomato vascular tissues against this pathogen [65]. Through the pathogen inoculation experiment, infection by *R. solanacearum* was found to induce substantial lignin deposition in tomato roots, suggesting that lignin may restrict pathogen spread within the vascular system by forming a physical barrier. Molecular analyses revealed that the receptor-like kinase FERONIA (*FER*) negatively regulates lignin deposition and plant immunity. Yeast two-hybrid assays confirmed that FER interacts with the NAC transcription factor *RD26* (Responsive to Desiccation 26) and suppresses its activity via phosphorylation. Further studies demonstrated that *RD26* directly binds to the promoters of lignin biosynthesis-related genes to activate their expression. Genetic experiments conclusively established that the *FER-RD26* signaling pathway enhances plant resistance by modulating vascular lignin deposition, forming a physical barrier to inhibit pathogen spread in roots [65].

Lee et al. systematically dissected plant defense mechanisms against *Pseudomonas syringae* infection in *Arabidopsis thaliana* [68]. Pathogen invasion induced significant lignin accumulation at infection sites, forming distinct lignin deposition zones spanning 3–5 cell layers around the pathogen. Molecular profiling revealed upregulation of *MYB58/63*, key transcription factors that activate lignin biosynthesis genes. In myb58/63 double mutants, lignin deposition was markedly reduced post-infection, accompanied by a threefold increase in bacterial spread. High-resolution electron microscopy showed thickened cell walls due to lignin deposition, effectively impeding pathogen penetration, while lignified walls reduced flagellum-driven motility of *P. syringae*. Additionally, ferulic acid, a lignin degradation product, exhibited direct antimicrobial activity. These findings demonstrate that plants employ lignin-mediated physical barriers and chemical defenses to establish robust disease resistance [68].

Tomato varieties resistant to bacterial wilt respond specifically to infection by constructing a vascular structural barrier composed of a lignin-suberin coating and tyramine-derived hydroxycinnamic acid amides. In susceptible tomato varieties, overexpression of genes related to the lignin-suberin pathway can restrict the movement of Ralstonia solanacearum within the tomato plant, thereby reducing disease impact. Using root drench inoculation, plants were infected via the roots with a bacterial solution containing 10^7^ CFU of R. solanacearum or a control solution. Bacterial samples of 10^5^ CFU/g tissue were then collected from the transition zone between the main root and the hypocotyl (approximately 1 cm below ground). In the susceptible cultivar Marmande, the level of 10^5^ CFU/g tissue was reached four days after infection, whereas the resistant variety H7996 required nine days to reach the same bacterial load. Using UV autofluorescence (which indicates the presence of phenolic compounds), it was observed that in the resistant variety, the vessel walls, xylem parenchyma cells, and tracheids exhibited strong autofluorescence. Additionally, following infection, a lignin-suberin coating was induced in the xylem vessels, forming a barrier that restricts the movement of the bacterial wilt pathogen [69].

### 4.4. Xylem Development Defects Lead to Dwarfing of Plants

This section discusses how xylem development influences four key horticultural traits: lodging resistance (Section 4.1), fruit and vegetable flavor quality (Section 4.2), disease resistance (Section 4.3), and plant height/dwarfing (Section 4.4). As detailed in Section 2, gibberellin signaling plays a central role in regulating xylem development and plant height. Building on this mechanistic framework, this subsection focuses on how specific xylem developmental defects—particularly those involving secondary cell wall formation and vessel differentiation—contribute to dwarfing phenotypes in horticultural plants.

Plant height is a crucial agronomic trait in horticultural plants that directly impacts crop economic value. Research has shown that phytohormones and xylem development are two key factors regulating tree height in fruit crops [31,32]. Normal xylem formation relies on the synthesis and deposition of secondary cell walls [32]. In pear (*Pyrus* spp.), PbXND1 (XYLEM NAC DOMAIN 1) has been identified as a negative regulator of xylem development. It induces a xylem-defective dwarf phenotype through its interaction with PbTCP4 (Teosinte branched1/Cycloidea/Proliferating cell factor 4) [31]. Genetic analyses revealed that pbxnd1 mutants exhibit increased xylem width, while PbXND1 overexpression lines display reduced xylem width, smaller vessel dimensions, and significantly decreased plant height. Conversely, PbTCP4 overexpression lines show enhanced xylem development, with markedly increased xylem width, vessel size, and lignin/cellulose/hemicellulose content. In contrast, PbTCP4-RNAi plants exhibit opposing phenotypic traits. Mechanistically, PbXND1 suppresses PbTCP4 transcriptional activity by competitively binding to target DNA sites, thereby modulating xylem development [31].

Growth regulatory factors (GRFs), a plant-specific family of transcription factors, play crucial roles in plant growth and development [33,36]. Previous studies have shown that *GRF6* (Growth regulatory factor 6) regulates plant height by promoting gibberellin biosynthesis and signaling [70], while overexpression of *rGRF1* induces abnormal dwarfism [71]. Recent research in Populus has demonstrated that overexpression of *PagGRF11* (Growth regulatory factor 11) results in pronounced dwarfism, characterized by thickened stems and shortened internodes [71]. Histological analyses revealed significantly increased xylem thickness and elevated lignin/cellulose content in these overexpression lines compared to wild-type plants. Notably, the content of S-type lignin monomers slightly decreased in the overexpression plants, suggesting that *PagGRF11* may regulate secondary cell wall formation by modulating lignin biosynthesis pathways. The molecular pathways underlying wood development in poplar are highly conserved with fruit trees at the levels of hormone signaling, transcriptional regulation, and cell wall biosynthesis. Populus species serve as ideal model trees for these studies due to their fast growth, tractable genetic transformation, and shared regulatory networks (e.g., *VND-MYB* cascade) with perennial fruit crops. The rapid growth and rich genetic tools of poplar provide transferable molecular modules (e.g., the *VND-MYB* cascade) for deciphering secondary growth in fruit trees. Research on poplar significantly shortens the exploration cycle for trait improvement in fruit crops [72,73].

## 5. Prospect

Based on the gaps identified in this review, future research on xylem development in horticultural plants should prioritize the following directions.

1. Distinguishing primary versus secondary xylem in horticultural contexts.

As highlighted throughout this review, primary xylem differentiation (typical of herbaceous vegetables) and secondary xylem/wood formation (characteristic of woody fruit trees) are often conflated in the literature. Future studies should explicitly distinguish between these two processes when investigating molecular regulatory mechanisms. Comparative analyses between herbaceous and woody horticultural species within the same family (e.g., tomato vs. woody Solanaceae) could reveal lineage-specific and conserved regulatory modules.

2. Validating model-species findings directly in horticultural crops.

Most mechanistic insights into xylem development—including miRNA-target modules (e.g., miR165/166-HD-ZIP III, miR319-TCP) and hormone signaling pathways—have been derived from Arabidopsis, poplar, rice, and maize. Direct functional validation in horticultural crops remains limited. Future research should prioritize CRISPR-Cas9-mediated knockout or overexpression of key regulatory genes (e.g., VND7, MYB46, miR319) in representative horticultural species such as tomato (herbaceous vegetable), apple (woody fruit tree), and rose (ornamental).

3. Connecting molecular pathways with precise anatomical traits.

Current studies often report molecular changes (e.g., gene expression levels) alongside gross anatomical observations (e.g., xylem width), but detailed quantitative trait mapping is lacking. Future work should establish high-resolution phenotyping platforms to quantify specific xylem anatomical features—including vessel diameter, vessel density, inter-vessel pit membrane thickness, fiber cell wall thickness, and cambial cell layer number—and link these traits to specific regulatory genes. This will enable precise breeding for optimized xylem architecture.

4. Integrating xylem anatomy, hydraulic function, and stress responses.

The relationship between molecular regulation of xylem development and whole-plant hydraulic conductivity remains poorly understood, particularly in woody horticultural crops. Future research should combine transcriptomic/proteomic analyses with physiological measurements of hydraulic conductance, stomatal behavior, and embolism vulnerability under drought or salt stress. Such integrative approaches will reveal how specific xylem anatomical traits (e.g., vessel diameter, pit structure) influence stress tolerance and water-use efficiency.

5. Translating findings from model systems to breeding applications.

The ultimate goal of understanding xylem development is to improve horticultural crop performance. Future efforts should focus on: (i) identifying natural genetic variation in xylem-related genes within germplasm collections; (ii) developing molecular markers for xylem traits (e.g., lodging resistance, drought tolerance, dwarfing); and (iii) using gene editing to introduce beneficial xylem alleles into elite cultivars without compromising yield or quality.

## 6. Supplementary Instruction

### 6.1. Information Sources and Search Date

A systematic literature search was conducted across three electronic databases: PubMed (via NCBI), Web of Science (Core Collection), and GeenMedical (a Chinese biomedical literature database). The final search was executed on 14 July 2026. No language restrictions were applied during the initial search; however, only English-language publications were subsequently included. Additionally, we manually searched the reference lists of retrieved articles and performed citation tracking to identify additional.

### 6.2. Inclusion and Exclusion Criteria

Inclusion Criteria: (1) original research articles, reviews, and meta-analyses published in peer-reviewed journals; (2) studies investigating molecular mechanisms of xylem development (including cell fate determination, vascular cambium activity, secondary cell wall biosynthesis, lignification, programmed cell death, and stress-responsive vascular remodeling); (3) studies conducted in horticultural plants (fruit trees, vegetables, ornamental species) or established model species (e.g., Arabidopsis thaliana, Populus spp., rice, maize) when findings are directly relevant to horticultural contexts; (4) publications in English; (5) publication date between 1 January 2011, and 14 July 2026. Exclusion Criteria: (1) conference abstracts, editorials, commentaries, and letters without original data; (2) studies focusing exclusively on non-vascular plants or non-horticultural species without relevance to horticultural crop improvement; (3) studies reporting only descriptive anatomy without molecular or mechanistic insights; (4) non-English publications (except for critical seminal papers with available English translations).

### 6.3. Study Selection Process

The screening and selection process followed the PRISMA 2020 four-phase flow diagram. Two authors (L.Z. and M.W.) independently screened titles and abstracts, and subsequently assessed full-text articles for eligibility. Discrepancies were resolved through discussion with the corresponding author (S.F.).

### 6.4. Rationale for the Search Strategy

We acknowledge that the term “GeenMedical” may be unfamiliar to international readers. GeenMedical is a widely used Chinese biomedical literature platform that integrates PubMed-indexed articles with Chinese-language journals, providing enhanced access to horticultural research published in China. Its inclusion ensures comprehensive coverage of regionally relevant studies that may be underrepresented in Western databases. The 17-year publication window (2011–2026) was selected to capture the most recent advances in molecular xylem biology while including foundational studies that established current paradigms. We explicitly distinguish between mechanisms validated directly in horticultural species and those inferred from model organisms.

## Figures and Tables

**Table 1:** Summary of evidence sources for molecular mechanisms regulating xylem development in model plants and horticultural crops.

Regulatory Category	Specific Mechanism	Evidence Strength in Model Species	Direct Evidence in Horticultural Crops	Primary Horticultural Categories
Hormone signaling	Auxin (ARF6/8) regulation of xylem differentiation	(Arabidopsis, poplar)	(tomato, apple)	Vegetables; Fruit trees
	Gibberellin (GA) promotion of cambium activity	(Arabidopsis, poplar)	(tomato, cucumber)	Vegetables
	Cytokinin (ARR) regulation of vascular differentiation	(Arabidopsis)	(tomato, apple, cucumber)	Vegetables; Fruit trees
	Brassinosteroid (BZR1-VND7) regulation	(Arabidopsis)	(tomato)	Vegetables
	Ethylene (RhPMP1-RhAUX2) regulation	(Arabidopsis)	(rose)	Ornamentals
	Abscisic acid (ABA)-induced lignification	(Arabidopsis)	(apple rootstock)	Fruit trees
miRNA regulation	miR165/166-HD-ZIP III	(Arabidopsis)	(pear, apple)	Fruit trees
	miR319-TCP	(Arabidopsis, poplar)	(tomato)	Vegetables
	miR857-lignin synthesis	(Arabidopsis)	-	-
	miR396-GRF	(Arabidopsis)	-	-
Secondary wall biosynthesis	NAC-MYB regulatory network (VND7/NST/SND)	(Arabidopsis, rice, maize)	(tomato, apple)	Vegetables; Fruit trees
	Lignin biosynthetic enzymes (PAL, CCR, COMT)	(Arabidopsis)	(tomato, carrot)	Vegetables
Stress response	Drought-induced xylem remodeling	(Arabidopsis, poplar)	(tomato, grape)	Vegetables; Fruit trees
	Salt stress-responsive miR319a-PagHKT1;2	(poplar)	(tomato)	Vegetables
	Suberin barrier and drought tolerance	(Arabidopsis)	(tomato)	Vegetables

**Table 3:** Linking regulatory layers of xylem development to horticultural traits and practical outcomes.

Regulatory Layer	Key Components	Xylem Developmental Process	Horticultural Trait/Outcome	Relevant Section
Hormone signaling	Auxin (ARF6/8), GA, CK, BR, Ethylene, ABA, JA	Cambium activity, vessel differentiation, secondary wall thickening	Lodging resistance, dwarfing, stress tolerance	Section 2.1, Section 4.1 and Section 4.4
miRNA regulation	miR165/166, miR319, miR857, miR396	HD-ZIP III repression, TCP modulation, lignification	Xylem patterning, plant architecture, stress adaptation	Section 2.2 and Section 3.2
Transcription factors	NAC (VND7, NST, SND), MYB (MYB46/83), HD-ZIP III (HB8), SPL, GRF	Secondary wall biosynthesis, vessel differentiation	Stem strength, lodging resistance, dwarfing	Section 2.2, Section 4.1 and Section 4.4
Secondary wall biosynthesis	Lignin (PAL, CCR, COMT), Cellulose (CESA)	Lignification, cell wall thickening	Fruit/vegetable quality (e.g., carrot), disease resistance	Section 4.2 and Section 4.3
Stress-responsive adaptation	ABA signaling, miR319a, suberin, laccase	Xylem remodeling, vessel proliferation, barrier formation	Drought tolerance, salt tolerance	Section 3.1 and Section 3.2
Vascular immunity	FER-RD26 module, lignin-suberin coating	Physical barrier formation (tyloses, lignification)	Bacterial wilt resistance (tomato)	Section 4.3

## Data Availability

Data are contained within the article.
